# Photodetection Properties of CdS/Si Heterojunction Prepared by Pulsed Laser Ablation in DMSO Solution for Optoelectronic Application

**DOI:** 10.3390/mi14081546

**Published:** 2023-07-31

**Authors:** Fatemah H. Alkallas, Shoug M. Alghamdi, Ameenah N. Al-Ahmadi, Amira Ben Gouider Trabelsi, Eman A. Mwafy, W. B. Elsharkawy, Emaan Alsubhe, Ayman M. Mostafa, Reham A. Rezk

**Affiliations:** 1 Department of Physics, College of Science, Princess Nourah Bint Abdulrahman University, P.O. Box 84428, Riyadh 11671, Saudi Arabia; fhalkallas@pnu.edu.sa (F.H.A.);; 2 Department of Physics, Faculty of Science, Taibah University, Yanbu 46423, Saudi Arabia; smghamdi@taibahu.edu.sa (S.M.A.); esobhe@taibahu.edu.sa (E.A.); 3 Department of Physics, Faculty of Applied Science, Umm Al-Qura University, Makkah 24382, Saudi Arabia; anahmadi@uqu.edu.sa; 4 Physical Chemistry Department, Advanced Materials Technology and Mineral Resources Research Institute, National Research Centre, Giza 12622, Egypt; emanmwafynrc@gmail.com; 5 Physics Department, College of Science and Humanities Studies, Prince Sattam Bin Abdulaziz University, Alkharj 11942, Saudi Arabia; 6 Spectroscopy Department, Physics Research Institute, National Research Centre, Giza 12622, Egypt; 7 Department of Physics, College of Science, Qassim University, Buraidah 51452, Saudi Arabia; 8 Higher Technological Institute, 10th of Ramadan City, 6th of October Branch, 3rd Zone, 7th Section, 6th of October City, 10th of Ramadan 44629, Egypt; reham.rezzk@gmail.com

**Keywords:** laser ablation, nanomaterials, PVA, optical properties, heterojunctions

## Abstract

The high-quality n-type CdS on a p-type Si (111) photodetector device was prepared for the first time by a one-pot method based on an ns laser ablation method in a liquid medium. Cadmium target was ablated in DMSO solution, containing sulfur precursor, and stirred, assisting in 1D-growth, to create the sulfide structure as CdS nano-ropes form, followed by depositing on the Si-substrate by spin coating. The morphological, structural, and optical characteristics of the CdS structure were examined using X-ray diffraction, transmission, and scanning electron microscopy, photoluminescence, and UV-VIS spectroscopy. From X-ray diffraction analysis, the growing CdS spheres have a good crystal nature, with a high purity and desired c-axis orientation along the (002) plane, and the crystallinity was around 30 nm. According to optical characterization, high transparency was found in the visible–near-infrared areas of the electromagnetic spectrum, and the CdS spheres have a direct optical energy band gap of 3.2 eV. After that, the CdS/Si hetero-structured device was found to be improved remarkably after adding CdS. It showed that the forward current is constantly linear, while the dark current is around 4.5 µA. Up to a bias voltage of 4 V, there was no breakdown, and the reverse current of the heterojunctions somewhat increased with reverse bias voltage, while the photocurrent reached up to 580 and 690 µA for using 15 and 30 W/cm^2^ light power, respectively. Additionally, the ideal factors for CdS/Si heterojunction were 3.1 and 3.3 for 15 and 30 W/cm^2^ light power, respectively. These results exhibited high performance compared to the same heterojunction produced by other techniques. In addition, this opens the route for obtaining more enhancements of these values based on the changing use of sulfide structures in the heterojunction formation.

## 1. Introduction

Scientists and researchers are very interested in semiconductor nanostructure materials because of their unique characteristics, which relative to the base material, consisted of optical, mechanical, chemical, and electrical properties. Several semiconductor nanostructured materials have the potential to be employed in a range of sectors, including optoelectronics, sensors, catalysis, and biomedicine, in addition to being inexpensive, recyclable, and stable. Furthermore, due to their substantial surface area and the high proportion of surface atoms, which enable special optical and electrical characteristics as compared to the bulk state, metal oxide nanostructure materials have recently attracted a lot of interest. Due to this, many scientists are very interested in creating materials with the desired dimension at the nanoscale, which could be considered promising and effective for a variety of manufacturing and technological uses, such as catalysts, solar cells, photodetectors, chemical and biochemical sensors, renewable power converters, sensing applications, and corrosion resistance [1,2,3].

In order to assure the creation of a strong bond that will allow for the processing of the bonded interface with high strength, two smooth and clean surfaces must first be joined at room temperature before their bonding is heated up during an annealing process. In this condition, the attraction forces draw the two bodies together into intimate contact, allowing connections to develop across the interface. This phenomenon has received great attention in the fields of mircomechanics, microelectronics, and optoelectronics, especially when semiconductors are present in this structure. The integration of heterogeneous materials is one of the bonding approach’s greatest strengths. The discrepancy in lattice constants impedes the integration of different semiconductors via heteroepitaxial development. In recent years, with the aim of integrating photonic and high-speed electronics with advanced silicon technology, there has been an emphasis on fusing compound semiconductors with novel silicon circuits [4,5,6].

CdS is a semiconductor that belongs to the II-VI group and has a direct wide-band gap of 2.42 eV at ambient temperature. The presence of CdS in nanostructured materials are usage in a variety of nanoscale semiconductor devices [7,8,9]. This material is commonly used in transistors, photo and gas detectors, and sensors. Even though Si has an indirect band gap, the CdS/Si heterostructures may be very useful for future optoelectronic devices and the production of emitter transistors, for instance. As a result, at ambient temperature, the interaction or bonding energy is usually minimal. As a consequence, the binding strength is strengthened using a heat treatment. Annealing encourages desorption of surface atoms, such as hydrogen, and the outdiffusion of molecules trapped at the contact. In the case of hydrophobic bonding, annealing also promotes the creation of covalent connections between the connected surfaces, similar to solid-to-solid bonding.

To accomplish this, several processes, including chemical, hydrothermal, mechanochemical, laser ablation, sol-gel, and thermal evaporation, focus on material preparation at this size. In the instance of the chemical precipitation method, which involves wet chemical procedures, it is thought to be the most efficient option for reaching this scale. However, the reducing agent is usually used to complete the reduction stage. As a result, as a byproduct or contamination, the reducing agent might be deposited alongside the eventual producer of nanoparticles. Thus, the completed product has some raw material contamination. Therefore, a lot of research is focused on producing pure nanostructured materials to satisfy researchers’ interests. Pulsed laser ablation in a liquid (PLAL), one of the best technologies for handling this problem, may be used to get around this fundamental problem [10,11,12]. It cleared the way for the creation of nanomaterials both with and without stabilizers as well as for the synthesis of a broad variety of unique and entirely pure nanomaterials in a variety of solutions, including metal oxides, nitride nanocrystals, carbide nanocrystals, and nanocomposite materials. Additionally, it could create nanomaterials in a variety of morphologies, including nanoparticles, dots, nanowires, and nanoflowers. Therefore, significant attempts have lately been undertaken to create pure nanomaterials utilizing PLAL in a variety of sizes, shapes, and compositions [13,14,15,16].

According to the non-ideal interface, the characteristics of wafer-bonded p-n heterojunctions are significantly influenced. Herein, PLA in liquid media technique was used to produce sulfide nanostructure materials of CdS by the pulsed laser ablation method of Cd metal immersed in sulfur precursor solution, followed by making and interference with p-type Si (111) substrate by the spin-coating method to form a heterojunction for photo-detection applications. The novelty of this work appeared in using PLA of Cd target immersed in DMSO solution to produce CdS nanoparticles with high purity. After that, different techniques were used to investigate. Then, the I–V properties of the built detector CdS/Si heterojunctions were investigated in the dark and under illumination.

## 2. Experimental Work

### 2.1. Materials

Cd metal (Cd, purity ≥ 99.9) with the dimensions of 5 × 5 × 2 mm^3^ was purchased from Sigma-Aldrich, England. The used target was added in the ultrasonic for 30 min to ensure that the tablet had been thoroughly cleaned, followed by drying in the oven for 2 h at 60 °C. Dimethyl sulfoxide (DMSO) and Cetyltri-methyl-ammonium bromide (CTAB) were purchased from Sigma Chemical Company (St. Louis, MO, USA). Ethanol is obtained from El-Nasr Pharmaceutical Chemicals Company, Egypt. Each of the reagents was laboratory-quality and utilized without extra purification.

### 2.2. Preparation Method of CdS NPs

**Figure 1:**
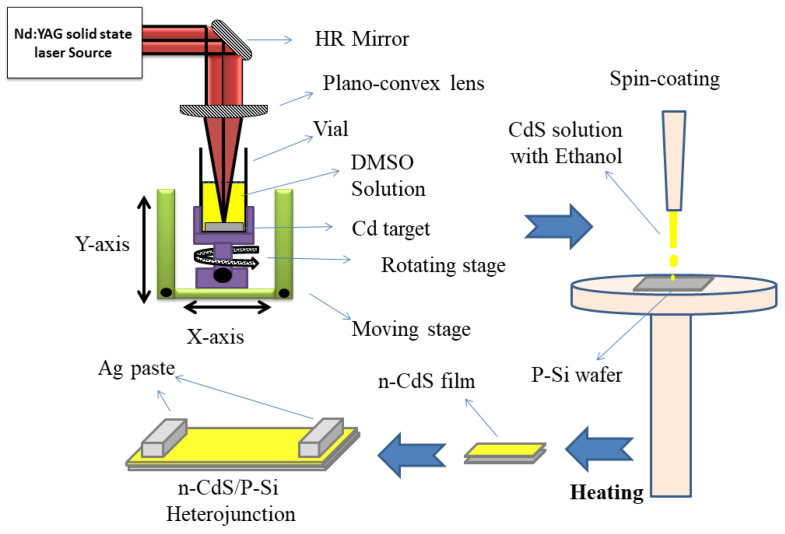
Schematic diagram of formation CdS/Si heterojunction via assisted pulsed laser ablation and spin-coating.

PLA of a cleaned metal Cd tablet in a liquid solution containing DMSO solution produced CdS nanoparticles. The target was positioned at the base of a glass container that contained 5 mL of DMSO. About 0.7 mm of liquid was present above the target surface. A 10 Hz repetition rate was set for Nd-YAG laser with the fundamental wavelength and a 7 ns pulse length. The target was exposed to a 60 mJ laser pulse. In order to alter the diameter of the CdS nanoparticles that were generated and focused by a convex lens (f = 70 mm). Ten minutes were spent doing the ablation. Following the end of the reaction, the resultant solution had been centrifuged and washed with ultra-pure water and ethanol to remove unreacted species and byproducts. Figure 1 depicts a schematic representation of this procedure.

### 2.3. Preparation Method of CdS/Si Heterojunctions

A uniformly dispersed thin layer of nanoparticles was created for the detector by spin coating CdS NPs on the surface of crystalline p-type Si (111) with an electrical resistance of 3–5 Ω·cm. The substrate was treated with an HF:H_2_O (1:10) solution and then washed with deionized water to remove the native oxide from the material. In order to create a homogenous solution, CdS NPs were dispersed in ethanol for two hours while being stirred at 60 °C. The pre-cleaned silicon wafer was then spun at 3000 rpm for 20 s to generate a thin layer of CdS film (area: 1.5 cm^2^). The solvent was then removed from the film by heating it to 300 °C for 15 min. For thickness measurement, the CdS layer has a thickness of 300 nm, as determined using a laser interferometer. Then, the contact electrodes were created using the silver paste (Figure 1).

### 2.4. Capacitance–Voltage Measurements

Ohmic connections were constructed by depositing films on Si wafer’s rear surface as well as on CdS NPs and Al thick films. To examine the conductivity type and mobility of CdS layers, hall measurements were made. Applied electrometer, power supply, and halogen bulb measurements of the dark current-voltage of the produced detector (CdS/n-Si heterostructure) were also made. On an HP 4294A RCL meter, measurements of the capacitance-voltage of heterojunctions were made at a frequency of 100 kHz.

### 2.5. Characterization Techniques

A UV-VIS-NIR spectrophotometer (JASCO 570, JASCO Inc., Hachioji, Japan) was used to study the optical properties (transmittance, absorbance and reflectance). Transmission and scanning electron microscopes carried by JEOL-JEM-1011 (JEOL Ltd., Akishima, Japan), and PHILIPS/FEI QUANTA 250 (FEI, Brno, Czech Republic), respectively, were used for studying the morphology. X-ray diffraction (Schimadzu XRD 7000, Shimadzu, Kyoto, Japan) was used to study the structure form. Energy dispersive X-ray spectroscopy (EDX) was used to analyze the elements utilizing TEAM^®^ software (https://teamsoftware.com/).

## 3. Result and Discussion

### 3.1. Physicochemical Investigation

Scientists were able to discriminate between CdO and CdS nanoparticles using their naked eyes in the ablation process of ultra-pure water, which exhibited a dark muddy white coloring for the former of CdO and a very faint yellow coloration for the latter. The colloidal solution made here has a somewhat different color than colloidal solutions prepared in water or DMSO, illustrating that the kind of solution influences particle size. Furthermore, the formation of turbidity color during the ablation of a Cd target in ultra-pure water demonstrates that nanostructured materials were efficiently constructed to prevent the formation of conflicts between the colors of DMSO and CdS NPs.

**Figure 2:**
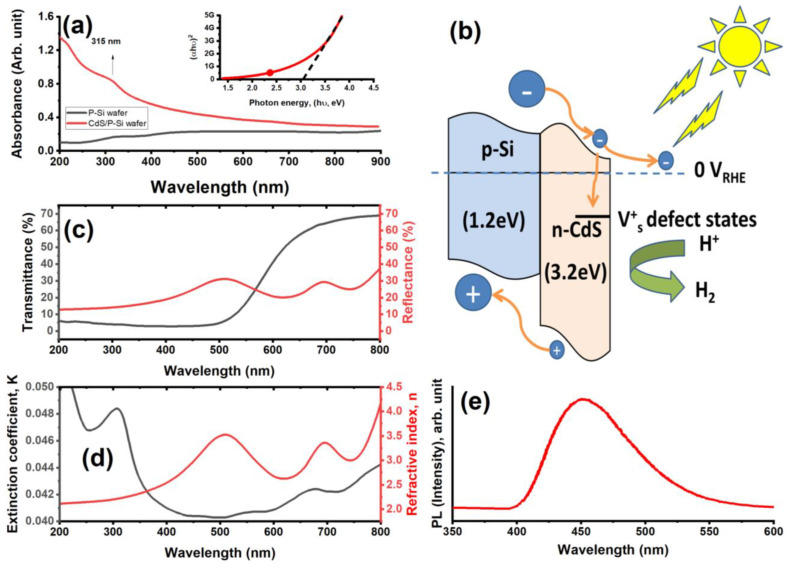
(**a**) The UV–visible absorbance spectrum of P-Si and CdS/Si heterojunctions associated with the direct transition of CdS/Si heterojunctions, (**b**) schematic of energy band diagram of CdS/Si structure, (**c**) transmittance and reflectance, (**d**) reflective index and extinction coefficient, and (**e**) the PL intensity of the prepared CdS nanoropes.

The UV–visible absorbance, transmittance, and reflectance spectrum of CdS NPs is depicted in Figure 2. As seen in Figure 2a, the transmittance and reflectance spectra showed that the absorption edge was found to be around ~315 nm. This peak conformed that their UV and visible-light activity is shown by the existence of an excitonic peak in the UV region and a lengthy absorption area [17]. In contrast, Figure 2c demonstrates that CdS films have an optical transmittance of 60% at wavelengths greater than 650 nm. However, optical transmittance did not substantially vary below 500 nm. Additionally, the prepared nanoparticles have a high absorbance at wavelengths less than 400 nm and a low absorbance at wavelengths above 400 nm, indicating the formation of smaller size particles [18]. In addition, CdS has a significant transmission in the visible range, making it suitable for use in solar cells and smart windows. Furthermore, the optical band gap (E_g_) of the prepared CdS NPs was visually assessed via Tauc’s law, (αhυ)^n^ = A(hυ − E_g_), where α, A, n, and hυ are the absorbance, the directly proportional constant, type of transition (2 for transition with direct property), and the value of energy band gap, respectively [19,20], which show 3.2 eV in its direct transition energy band gap. Thus, from Figure 2b, the CdS/Si electrode, surface defects on the electrode-electrolyte interface may capture photoelectrons because of defect energy levels that exist above the conduction band of CdS, leading to carrier recombination at the electrode–electrolyte junction and increased hydrogen generation performance when lighted [21,22]. Furthermore, from Figure 2d, the extinction coefficient (k) and refractive index (n), which are important for optoelectronic applications, were also computed using the following formula:

$$n=\frac{1+R}{1-R}+\sqrt{\frac{4R}{{\left(1-R\right)}^{2}}-k^{2}},\;k=\frac{\alpha \lambda }{4\pi },\;\alpha =2.303\left(\frac{Absorbance}{thickness}\right)$$

where α is the absorption coefficient, λ and R represent wavelength and optical reflectance, respectively. As seen from Figure 2c, k values abruptly dropped down below 0.041 for λ > 400 nm, leading to n values in the range between 2.08 and 4.15 for the wavelength range of 200–800 nm. Additionally, PL spectra for the created CdS nanostructured materials were obtained as shown in Figure 2e. A sharp peak at 546 nm in the PL spectra of the films produced at 449 nm, respectively, indicate the band-to-band transition and defect-related emissions [23,24].

**Figure 3:**
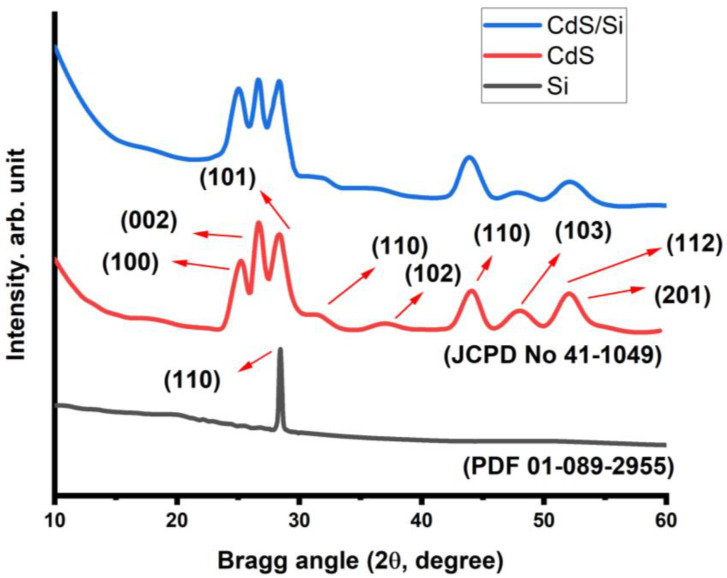
XRD diffractogram of CdS nanoropes, Si wafer p-type, and CdS film on Si (111) substrate.

Figure 3 shows the performance of the XRD patterns of the formation of CdS NPs, Si wafer p-type, and CdS film on Si (111) substrate. In the pattern, the diffraction angles of 25.18°, 26.61°, 28.32°, 31.46°, 44.03°, 48.31°, 52.16°, and 52.88° are delegated to the planes of (100), (002), (101), (110), (102), (110), (103), (112), and (201), respectively, for the hexagonal structure of CdS based on (JCPDS N. 01-0780). It denotes wurtzite structure and c-axis orientation of the developed CdS film with respect to the Si (111) substrate. The clarity of the XRD peaks indicates that the particles are metal NPs in their crystalline state. The absence of additional impurity peaks in this spectrum shows that the NPs generated under these preparation circumstances are pure. The FWHM readings would change slightly because of the variable crystalline grade [25,26]. The Debye–Scherrer equation was used to determine the crystallite size:

$$D=\frac{0.9\lambda }{\beta \cos\theta }$$

where β is FWHM and θ is the Bragg angle. The value of the CdS NPs crystallite size at the Bragg angle of 26.58° was found to be about 24 nm. These values are somewhat consistent with the calculation value from the absorption on the crystallite size.

**Figure 4:**
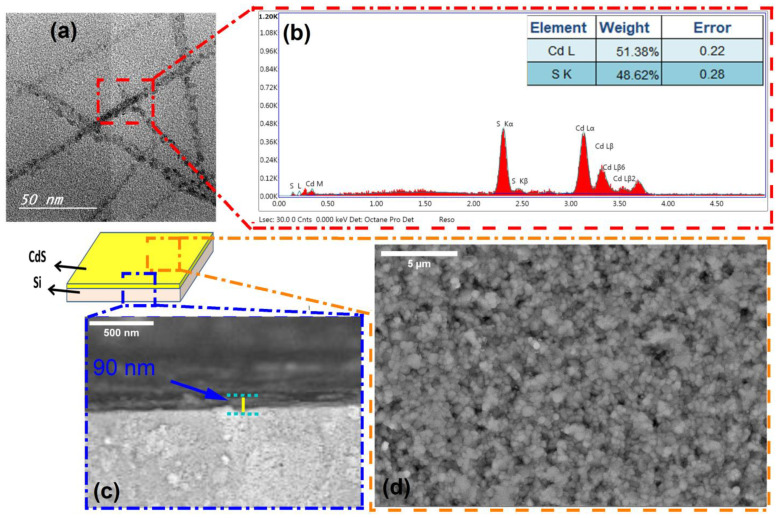
(**a**,**b**) TEM image and its EDX analysis of the prepared CdS nanoropes and (**c**,**d**) schematic representation and SEM image of CdS nanoropes deposited on the Si substrate from thickness and upper surface.

The TEM visualization and size variation of the produced CdS NPs are shown in Figure 4a. The produced nanoparticles are approximately spherical with a size of roughly 23 nm and aligned to look like ropes. Additionally, the EDX patterns for the prepared CdS NPs (Figure 4b) showed only S and Cd without presence of C and O atoms appearing due to the high purity of the PLAL method and a good washing and removal of the residual contaminants from the DMSO solvent. The elements of cadmium (Cd) and sulfur (S) were discovered, which confirmed the successful formation of the CdS structure. Furthermore, the CdS layer was formed on top of the Si substrate with good adhesion, as shown in the schematic construction of the CdS/Si photoelectrode. To achieve a continuous CdS layer formed across the whole Si substrate, the influence of deposition time on the thickness and shape of CdS was investigated, as seen by SEM images in Figure 2c,d. There are no visible pinholes, cracks, or colloidal precipitates in the film, as seen by the SEM image of the CdS surface (Figure 2c). In addition, the CdS is created that is around 90 nm thick, as seen by the cross-sectional SEM picture (Figure 2d).

### 3.2. Optoelectronic Study

**Figure 5:**
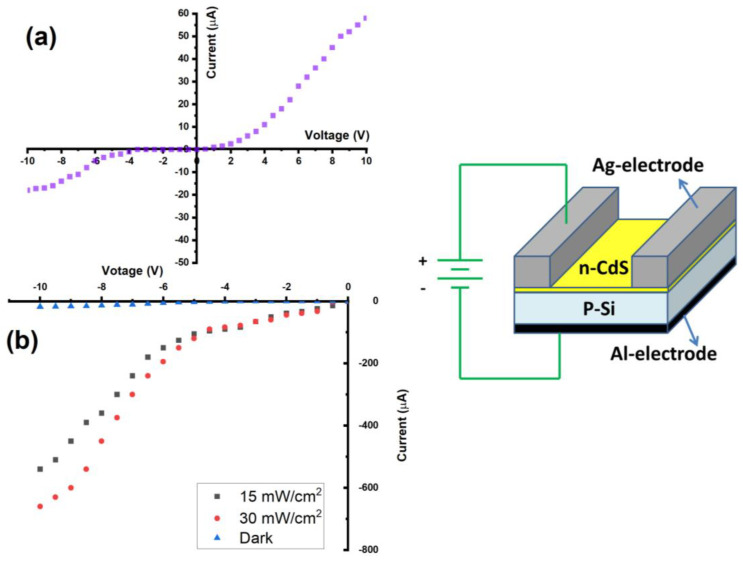
(**a**) Dark current and (**b**) photo current of CdS/Si heterojunction.

The built photodiodes’ I–V properties were examined in both dark and lit circumstances. All measurements were carried out at room temperature. The high recombination was shown to result from the bonding p-n Si/CdS heterojunction in free air and the following high-temperature annealing. Figure 5a displays a dark current-voltage measurement of the Si/CdS heterojunction photodetector. The photodetectors display rectification behavior, which denotes the presence of a connection between the silicon substrate and the CdS layer [27,28]. It was found that the current, which is typically referred to as a recombination current, rose exponentially with voltage larger than 2 V in the forward bias owing to the reduction in depletion layer width. However, the magnitude of this current does not vary noticeably at voltage below 2 V. The forward current is constantly linear, while the dark current is around 4.5 µA. Up to a bias voltage of 4 V, there was no breakdown and the reverse current of the heterojunctions somewhat increased with reverse bias voltage, while the photocurrent reached up to 580 and 690 µA for using 15 and 30W/cm^2^ light power, respectively. The ideality factor (β) of the heterojunction was determined using the following calculation using the diode equation [29,30,31].

$$\beta =\frac{q∆V}{KT\ln\frac{∆I}{I_{s}}}$$

where q is the charge, K is the Boltzman constant, T is the used temperature, and I_s_ is the saturation current, as shown in Figure 5. Thus, the values of β for Si/CdS heterojunction were 3.1 and 3.3 for using 15 and 30 W/cm^2^ light power, respectively.

In contrast to a dark environment, Figure 5b depicts a typical I–V characteristic for a Si/CdS heterojunction under light (15 and 30 mW/cm^2^). Without any saturation breakdown, the photocurrent was silently raised with bias. Additionally, the photocurrent rose as illumination increased because more e-h pairs were formed as illumination intensity increased. This helps to explain why the photocurrent for the identical heterojunction device is larger than the dark current. This outcome suggests that the manufactured photodetectors have excellent linearity properties. Additionally, this study shows that the CdS considerably enhanced the photodetection capability and merit figures of the CdS/Si photodetector [28,32,33].

## 4. Conclusions

In this study, the pure Cd target in DMSO solution was effectively ablated with a pulsed Nd: YAG laser to create CdS NPs. The production of cubic structures in CdS NPs was demonstrated by XRD measurements. The optical band gap for the UV–visible absorbance spectrum is roughly 3.2 eV, with the absorbance peak occurring at 400 nm. As a result of the quantum confinement effect, this is superior to the base material. Last but not least, the Si/CdS photodetector’s features and performance demonstrated that the junction exhibited proper rectification behavior. Hence, the CdS NPs may therefore be a good candidate material for optoelectronic applications. These results indicate that the photodetectors produced have outstanding linearity qualities. Furthermore, this work demonstrates that CdS significantly improved the photodetection performance and merit figures of the CdS/Si photodetector.

## Data Availability

Not applicable.
